# The Work of the Hospitals

**Published:** 1892-06-11

**Authors:** 


### The Work of the Hospitals.

Year by year, as Hospital Sunday comes round once more, we ask ourselves the question: How can we best put the work of the hospitals before our readers, and before the preachers whose business it will be to urge upon their hearers the claims of these standing witnesses to the humanity and Christian sympathy of certain members of the community—alas! that we cannot say of all. What we want is to see the hospitals as the patients themselves see them; and with that object in view, we have this year called together a little band of "faithful witnesses," who have had ample opportunities of learning just what we want to know. It will be seen that, as far as possible, we have gone to the patients themselves for information. There are large numbers of men and women who know the insides of many more hospitals than one, though the case of Catherine Burt, whose testimony will be given later on, is a rather exceptional one. She is only about five-and-forty now, but years ago she told us laughingly that she believed she had been in every hospital in London excepting one, and that she really thought she must some time try it—the "Westminister," as she called it —a not infelicitous rendering of the name, by the way. It would be really difficult for anyone but a doctor to think of a disease which she has not had, and her family history, when you come to hear it, convinces you that she comes of a stock with "no constitution"; but she is wonderfully bright, and earns her own living as a hard-working and most willing general servant—when she is not in hospital.

Thomas Atterton, again, a very refined-looking and intelligent man, who has worked very hard as a draper's assistant—long, in fact, after he ought to have taken to his bed, has been struck down by various illnesses several times, and will never, perhaps, be really strong again. We saw him first in one of the wards of a large London hospital, where, it is to be feared, he will yet be when these pages are printed, and were struck by the keenness of his eye, and the intelligent interest with which he noticed all that went on around him.

Then, as we cannot expect of children a good all-round view of children's hospitals, however many they may have been in—and some of the poor little things have seen not a few—we ask Mrs. Wilkins, a poor woman who has had two or three of her children in hospitals at various times, to tell us something of what she has seen there; while to her account are appended a few remarks by Miss Mather, a lady who does "outside work" for a hospital for that sad scourge of child-life, hip disease, and whom we have asked to tell us something of the chronic diseases of children, and incurable cases, and of what may be called the supplementary work of the hospitals—the help given to needy patients through Samaritan funds and convalescent homes.

We also put before our readers a statement, by no means exhaustive, of the benefits which the fairly well-to-do receive, or may receive, from the hospitals, given by a medical man who has sent two or three of his own children to be nursed at the Fever Hospital of which he speaks, and thus gives practical proof of his satisfaction at the way in which it is conducted.

It is hardly necessary, perhaps, to say that these accounts of hospital life are not to be taken *cum grano;* they have been carefully verified, where verification has been necessary, and all who read them may take them as simple statements, not of what might be, but of what is.

### I.—HOSPITAL ACCOMMODATION FOR BREAD-WINNERS.

Thomas Atterton *loquitur.*

"Yes, sir, I quite understand that it is not so much me as the hospitals you want to hear about. I've been in three altogether, counting this one; that is to say, I've been in a hospital for accidents, down in the East-end; a general hospital—that's this one; and a special one, as I suppose you call it—a Hospital for paralysis, and fits, and things like that. And you'd like to hear a bit about them all, you say.

### "Broken Legs, Broken Ribs, Broken Everything."

"Well, it wasn't for an accident that I went into that hospital in the East-end. I was working down that way just then, and I had a pretty bad abscess—you know, sir, I never was a strong chap; and my doctor was one of the hospital doctors, so he got me in there, saying I should be seen to much better than I could be at home. But all the other chaps were in for some accident or other—it's a terrible place for accidents, that part of London, down by the docks. We'd have men brought in with broken legs—I'd be afraid to say how many we had in with broken legs all at one time—broken ribs, broken heads, broken everything. And then the flesh wounds—I've seen a poor fellow with the side of his head regular ripped off; and another—a railway porter he was—with his arm looking all torn to bits. He was brought in one night about eleven o'clock, I remember, and for three hours there were three doctors round him, as well as the nurse and the porter. Presently two of the doctors came up to wash their hands close by me, and I heard one of them say, 'We'll try to save the arm, after all, I think. It'll be damaged, of course; but a damaged arm's better than no arm at all.' And so they did save the arm, and the poor chap got better. There was a little lad come in who'd got his leg right up to the knee into a lot of boiling tar; they put *him* all right again, too. Of course, there was a lot that died—they were too badly hurt to have any chance, some of them; but if they died, it wasn't for want of looking after, I know that. There didn't seem to be any trouble that nurses and doctors weren't willing to take; and when I asked if I might be put on a different diet—for, you see, I wasn't like most of the other men, right enough in health except for the broken bones, and with appetites like ostriches, as you may say—why, they attended to it right off. I never had to ask again.

### In a General Hospital.

"But about this hospital, sir. Well, I've been in here a long time now, for you see there've been bits of diseased bone to come away out of my arm, and altogether it's been a bad case. But I've never felt dull. The nurses 'll give us little jobs of work to do for them —hemming dusters, and turning sheets, sides to the middle—you know how they do it, sir. And then, the other patients—why, there's always something to see or hear about them. That man with a bad leg weighs twenty-one and a half stone; it's a good thing he can help himself a bit. And that little chap there came from Wales; he cried a good bit at first, but now he's as happy as a king, spite of knowing that he's got another operation coming on.

### Bronchitis and Starvation.

"You see that old man there, sir—Daddy, the nurses call him. He came in with bronchitis, but he was pretty near starved, too, they say. He'll do anything for the nurses, he's so grateful to them; and they've hard work to keep him from tottering about on his weak old pins trying to save them a step or two. It's my belief, sir, that they've hidden him away in the ward-kitchen once or twice, for fear the doctors should see him and say he's well enough to go out. He won't hear of going into the House, though he really isn't a bit fit to work, and I know they think he'll be be found dead some day of sheer starvation, poor old chap.

### Mikie, the Pet of the Ward.

"Then we've got four cots in the ward, sir, and they're a great interest to us. I don't think any of us 'll soon forget little Mikie—he was over there in that cot for months. A funny-looking little chap he was, only four years old, with a surgical jacket on that pushed his little chin right up in the air. We'd all of us have done anything for Mikie, with his pretty, winning little ways. All the chaps that could get about would go and wait on him, as if he was a king; and there was one of 'em that wasn't allowed to be out of bed for more than an hour at a time, who would always get up to feed Mikie with his breakfast, and then go back to bed again. I can't tell you what we felt when Mikie got worse. We couldn't think of anything but him, poor little chap, as he lay dying there; and when one day the screens went round his cot, and nurse told us Mikie was dead, I don't believe there was a man in the ward who didn't feel as if he'd lost a child of his own.

### One Taken; the Other Left.

"But we've got little Tommy yet, and he's been a great comfort to us all. You can see him now, sir, playing with that thin-faced young fellow over there. That's a medical student, who's been warded for two or three weeks now. For ever so long they lay in beds next to each other without saying a word—at least, Tommy never did, he was so shy. But he lay looking at and admiring Mr. Allard all the time; and at last he took to writing him letters, and handing them to him through the rails of his cot. How we did laugh over the first one! 'Dear Mr. Allard,—Are you going to shave this morning, and me hold the glass? How many brothers and sisters have you got, Mr. Allard?—I am, your Tommy.' After that, they got to be great friends, and they had their photographs taken together the other day.

### Pheasants for Dinner.

"You were asking me about the diet when you first came in, sir. Well, I should like just to say that I haven't the least word to say against it. It's always good and tasty; and now and then there's some game or something sent for us—I shouldn't mind if it was oftener. The other day someone or other sent twelve pheasants, I heard; anyhow, we had pheasant in here, and I can tell you it *was* good—brown gravy, and bread sauce, and everything, fit for a king to eat!

### No "Knicknacks" at Home.

"I tell you what it is, sir; I do believe it is often the food, as much as the nursing and doctoring, that picks a man up. How can lots of these poor fellows get the strengthening things they want, if they are nursed at home—let alone things to bring back their appetite when they've got none? I've often thought of a poor chap I knew when I was down in the East-end. He was laid on his back for ever so long, with a terrible abscess under his chin; and one day, when I went in to see him, and asked him how he did, he burst out crying (he was terribly weak, you see, sir), and he said, 'Oh; I think I should get on if only I had a few knicknacks, such as they give to sick people, but you see I can't get 'em here.' It's different down in the country, where there'll generally be a squire's or a parson's lady that'll run in to see you with jellies and beef-tea, and I don't know what all; but in these poor parts of London, where there isn't a rich man about—for you certainly can't call these East-end parsons rich men—the sick have a terribly poor time of it, there's no mistake about that.

### A Hospital for Nervous Diseases.

"But now I've got to tell you a little bit about that other hospital I was in—the one for paralysis and fits. Nervous diseases they're called, I hear; and I'm told, too, that nervous diseases are getting much more common now than they were—we're living so fast in these days. People with diseases like this generally want to be much longer in hospital than other patients do, and I suppose that's why there's a special hospital for them, where they can be kept for a year or two—sometimes, I believe, they're kept as much as four. There's a Pension Fund, too, to help patients who are found to be incurable, but I don't know anything myself about that, thank God for it! I wasn't in so very long, but I saw a good many cases of paralysis, and I must say the interest the nurses and doctors took in the cases was wonderful. I've seen the nurse flush up quite red with pleasure when she found that one of her patients, who had had no feeling in his legs for months, could just feel the prick of a pin. There was one poor young fellow who seemed to have lost all power in his mind as well as his body; he lay there in a little alcove pretty much to himself, never speaking or moving, and they used to feed him with spoonfuls of finely minced-up meat. I never heard if he got better, or if the man in the next bed to me did—a dock labourer who'd been in two years, and had a wife and two children at home. But they do have some wonderful cures there; they'll operate upon the brain or the spine, they tell me, and take away some little thing that presses, and then the patient 'll get right again in no time.

### "A Monster unto Many."

"Did you ever see a man come out with lumps all over his skin, sir, like the lumps on an old tree, as you may say? I've seen one or two like that in this hospital that I'm talking about—it sometimes comes on after fits, I've been told; but the worst case that ever I saw was a man that's always been out and about till just lately. He isn't like a man, sir; he's more like an old gnarled tree, or a rhinoceros, and yet that isn't lumpy enough—there's nothing that is, that I can think of. There isn't a smooth place on his body, nor yet on his head,—he's all masses of lumps; and what that poor chap has suffered, no one 'll ever know. His mother was good to him, he says, but his brothers and sisters would have nothing to do with him; he is hooted and followed in the streets; and when he once joined a temperance society, the members all said they would leave if he didn't, they couldn't bear the sight of him. But there's one place where they have been kind to him, and that's the hospital; he told me so himself. He's in a big London hospital again now, being treated for his eyes, for these lumps interfere terrible with his sight; and he says the patients, as well as doctors and nurses, are all very good to him. There's some talk of his being allowed a room there, where he can live, if the expense can be arranged for. 'I *should* be glad to be taken in,' the poor fellow says, 'like the elephant-man was, you know. The streets isn't the place for me; and yet, if I live out, I must go about selling wood, or how can I get my bread?' I do hope it 'll be arranged for him somehow or another.

### II.—HOSPITAL ACCOMMODATION FOR WOMEN.

### Catherine Burt's Experiences.

"I ought to be able to tell you about hospitals, if anyone can, seeing the number I've been into. And there isn't one of 'em that I haven't liked; I may as well say that straight away. The doctors have often said to me, 'Why, you ought to be a nurse yourself!'—I know such a deal about diseases and complaints, my own and other people's. Yes, and I've seen an operation, too; it was in the next bed to mine, and I peeped round the screens and saw it—it was an operation on the breast. Silly of me? Yes, I believe it was; and it isn't the only time I've been silly in hospital, either; for one day, when I was getting better from gastric ulcers, I got up, and went round to see all the other patients, and one of 'em (they all wanted to do something to show they was pleased to see me about again)—one of 'em gave me a bit of sponge cake with currants in it. I couldn't help eating it, though I knew I oughtn't to; and that night—oh! there *was* a to-do, me screaming with pain, and Sister and the doctor round me, and all of them saying 'She's been eating something; she must have been eating something!—'till I had to tell 'em right out. I'm not going to be so silly as that again, I can tell you!

### Sad Cases.

"I don't know if you'd call it silly too to be upset about the other patients, but I can't help it sometimes. I've seen a girl brought in on a stretcher late at night, who'd been crossed in love and poisoned herself; and another with an abscess in her throat, whose screams were awful to hear—she died in two hours. There was another poor woman, who had something the same; it was strange how I met her twice. She was quite blind, and a good-living woman, she was; and once when I was down at a convalescent home, after one of my visits to hospital, I met her there, and I used to read to her. When we left, I never thought I should see her any more; but one night when I was in hospital again—a new one this time—I was talking away to my neighbour, and suddenly I heard a voice from a bed a new patient had just come into. 'Is that Kitty?' it said; and there was my blind friend again. Poor thing! she never came out no more. She had a dreadful tumour in her throat, where that brute of a husband of hers had hurt her (he always would go for her throat when he was angry, she told me); and though the doctors put a tube in her throat, it wasn't much good; the tumour was too low down, and she couldn't get her breath. So at last she was choked, poor thing! But her last hours was as happy as they could be, considering what she had to bear. Whatever that poor creature 'd have done without a hospital to come to, I'm sure I can't think.

### Broken Bones.

"You'd be surprised to see how quickly old people get over accidents in hospital. Once we had three broken thighs in the ward at the same time, all slung up in the air—the way they treat them at that hospital. They were all old women, and one of them was eighty-five, and she had a bone in her arm broken too, and bronchitis, so that a kettle had to be set steaming into a tent they made for her, night and day. Then we had a woman with a broken leg—she was there already when I came in (I'd got drinking diabetes that time); and when I was helping the nurses that first day by pinning up the counterpanes—as soon as ever I got to her, she shouted out, 'Don't you come near me!' 'Why, what's the matter with you?' I says. 'Matter!' she says, 'I was just getting better from fever, and I got out of bed, and if I didn't slip down and break this old leg, and that'll keep me here a good three months more, I expect! Matter, indeed!' and she turned away as cross as two sticks.

### Startling the Students.

"I've seen several of 'em terrible cross; perhaps their illnesses made 'em so. It was just wonderful how the nurses would put up with their tempers, trying to soothe 'em all they could. I've seen a woman with heart disease, who'd snap your head off if you did but ask her how she did. 'What's the good of asking me how I am?' she'd say. 'How do I *look*?' Then she'd say, quite loud out, 'Here's that silly old doctor coming round again!' or 'Here's that old cat!'—meaning the nurse. But they were as kind as kind to her, and I've een the nurse stand over her for I don't know how long, trying to coax her to eat something. I did laugh once at the way she frightened two young doctors. They'd all gone past her that morning, thinking she was asleep, and they wouldn't worry her, when two of 'em slipped back on the quiet just to listen to her chest for a minute. She lay still enough till they'd begun, and then she suddenly burst out in a voice fit to wake the dead, 'Take your great fat heads off my chest, will you?' You should just have seen 'em start! She died while I was there, but I believe she did see, before she died how good they'd all been to her, only her temper was that bad she couldn't control it.

### Greedy Grumblers.

"Some of them were pretty greedy too. They'd be put on nothing but cold milk, perhaps, when they were very bad, or plain milk pudding, or 'Brown essence,' as they'd call it, and they'd grumble dreadfully—in fact,

I'm sure they'd have had a poor chance of getting better if they hadn't been in hospital, for they'd have got hold of other food directly if they could—not to speak of beer and spirits. There was a poor young girl of fifteen or so come into hospital, when I was there, pretty well starved; she'd four little brothers and sisters, and her father had been out of work I don't know how long. Well, there wasn't a great deal the matter with her except starvation; and she'd do a lot of little jobs for us, running round and tucking in the bed-clothes, and saying, 'You'll ketch cold; why don't you keep yourself kivered up?' and fetching us that precious little hare-lipped baby that we all wanted to have a hand in nursing, or bringing us our dinners. Some of them would try quite hard to get her to bring them bread, or something else they mustn't have; and when she wouldn't, I've heard them say, 'No, you greedy gut, you want it yourself!' And there was one woman there who wasn't ashamed to ask to change her rice-pudding for the custard that was ordered for the next patient—quite the lady, she was; she'd been a governess, I think. And the poor lady did it for several days; she didn't quite like to say no, you see, though she'd ought to have done it, of course. Still, there was a great many that was as gentle and grateful as could be; and those that were rough and rude—well, they was pretty nearly always better behaved before the time came for them to go.

Two Chest Hospitals.

"But I've told you nothing yet about the Chest Hospitals I've been to; they're mostly for long cases that can't be taken into the general hospitals, you see. I was in both of them for a good long time, and, of course, I've seen plenty of poor things that have been in for years. But you see people get better in these hospitals that you wouldn't have thought could ever be well no more; the care and attention they get, and the good food, and the malt and cod liver oil—they use ever so much of that—make different women of them —and men, too, I've heard say. Of course, I don't know anything about the men's wards, but I heard the doctor say one day, when I was there, that they had such a lot of clerks as patients—quill-drivers he called them. 'Oh, dear me! dear me!' he says, 'why will all these chaps take to office stools, instead of working with their hands, and breathing in the fresh air? Black coats and white hands—that's all they think about nowadays!'

"You Must Come in."

"I hadn't no thoughts of going into the Chest Hospital that first time, you know, sir; I only went as an out-patient. But when the doctor tried my chest, he looked as grave as grave, and he said, 'You must come in, you know;' and when I said, 'I don't want to, sir,' he says, 'You must. Do you think you know better than I do?' He gave me a letter straight away—he was that troubled about me; and I had to go in—a gathering on my lung, I think it was.

A Lying-in Hospital.

"I can't but say a word, if you'll let me, sir, about lying-in hospitals too, though, of course, I never was in one, except on a visit to my poor sister that's gone. She went into one, time after time—most of the women who've been there once go again, they tell me; and some of them are only too glad to pay whatever they can afford, for the privilege of being nursed there. When I went to see my sister there once, she told me 'it was like a fortnight of heaven.' They didn't take none but married women there, except just once in a way a very special case; but, of course, there are other lying-in hospitals that do. Oh, but I was sorry for some of the poor women there! What they'd have done without such a place, I don't know. There was one, the wife of a marble polisher, who'd gone stone-blind, poor fellow; and just a month or two after that, her time came, and she had twins. And there was a poor woman who had walked all the way from Poplar to the hospital—which is right in the heart of London, as you may say—because she had no money, and I don't suppose the trains were running so early, neither, for she started at two on Sunday morning, and got there at eight, stopping now and again, because she was too bad to walk. Then there was another poor woman—her baby was born with a dreadful place on its back, and there wasn't but one position they could put it in which didn't hurt it. It had two nurses to see to it, and take it on their laps in just the right way; but it was dying fast, they told me. 'However did it get like that?' I asked; and the Matron, a dear, kind woman, said, 'Oh, a fall, or something!' But when we'd got out of hearing of the poor mother, she whispered to me, 'Her husband kicked her; *that's* what it was!'

"I'll Teach 'em!"

"The mothers are churched, and the babies christened, before they go out; and as for the disinfecting that goes on—why, you'd think it was a fever hospital, from the pains they take; but that's all as it should be, I know. I shan't soon forget how comfortable and cosy everything looked. And when I said to the Matron, 'I should think there's none that ain't happy here, mum,' one of the women got right up in her bed, and said, quite fierce-like, 'If anybody's got a word to say against this 'ere 'orspital, let 'em come and say it afore me—*I'll* teach 'em!'"

---

### III.—WHAT THE HOSPITALS DO FOR THE MIDDLE CLASSES.

By a Doctor.

"I am not at all sorry to have the chance of saying a few words about what our hospitals do for the middle classes—and, I might add, for the upper classes; for of this I am sure, that most fairly well-to-do people think of hospitals as useful merely to the lower strata of society, except perhaps as a temporary refuge in cases of sudden illness or of accident. There is a very large class, however, to which the hospitals are far more than this—I mean persons of limited income, to whom a long illness at home means terrible distress and straitening of means, and perhaps even beggary. What this class would do without our consumption hospitals and our hospitals for nervous diseases, where patients may be kept for two, three, or even for four years, I cannot conceive. In them you will find poor governesses, clerks, professional men, tradesmen who have struggled on, Heaven knows how, to support themselves and relatives dependent upon them, and have at last utterly broken down.

Clerk by Day, Journalist by Night.

"Take, for example, a poor foolish fellow, whom I saw the other day—and yet, who knows what the pressure of circumstances may have been upon him? A clerk by day, a journalist by night, he toiled on month after month; then at last the inevitable breakdown came, and now for the best part of a year he has lain there, a helpless inmate of a hospital ward. It goes without saying that the guinea a-week which such patients pay, if they can, nothing like covers expenses. Surely those who know that, if overtaken by illness, they would need this help, should, while their means allow of it, do something towards keeping such hospitals up; while richer people may well feel that here is a claim on their charity quite as pressing as the claims of the so-called working-classes.

A Last Resource.

"I am often asked whether persons do not attend hospitals as out-patients who could really afford to pay for their treatment at home? Well, no doubt this may happen now and again; but we mostly find that our better dressed out-patients have had a long course of treatment at home before coming to us. I remember a man—a simple, countrified sort of fellow he was—whom we cured at our hospital, and who, after he was cured, looked quite weighed down with care, we couldn't think why. At last it came out. 'I've spent over £80 for medicines and things that haven't done me a bit of good,' he said; 'and whatever will they ask for *curing* me?' Another case, too, comes to my mind—that of a well-dressed lady whom I found sitting among the out-patients one day. Her eye had been bitten many years before by a wolf-hound, and she had suffered ever since, though she had been to see her oculist every fortnight since the accident happened. 'Can't attend to you, mum,' I said, looking at her shot-silk dress; 'starving patients waiting for me, you see.' But she begged and implored me to give my advice and help; I took out the offending eye, and her health was quickly restored. It must be remembered that to come for treatment as an out-patient means waiting about, often for hours, among all sorts and conditions of men, and various disagreeables which a person able to pay would generally rather not face.

A Hospital for Infectious Diseases.

"But here is yet another boon which our voluntary hospitals confer upon the middle and upper classes. There is an excellently-arranged fever hospital—or rather, a hospital for all infectious diseases but small-pox—in which Londoners living within a certain area (it has been decided for convenience sake, that this shall coincide with that dealt with by the Metropolitan Asylums Board) may be treated, on payment of three guineas per case, or, if they can afford it, three guineas a week. All arrangements are carefully thought out. There is a separate block for scarlet fever, another for measles, another for typhoid, and so on; and the best proof of the care with which it is managed is to be found in the fact that no patient has ever yet contracted another disease than that with which he was brought in. And as to the success of the treatment, I need only say that, while 388 cases of scarlet fever were treated there last year, only three patients died. We doctors find this hospital invaluable. I have sent my own children there, thus relieving my mind of much worry about them and about my own patients; and many doctors and nurses go in when ill, not to speak of various other persons who could not be nursed at home without great expense and great risk to others.

"How Do You Get Admitted?"

"Why, it is simple enough. Directly you find that your child, say, is suffering from an infectious illness, just get your doctor to give you a medical certificate, and send off to the hospital. In a very short time the hospital brougham-ambulance will be at your door, with attendants and a nurse, and away it goes. The little patient can be visited twice a week, due precautions being, of course, observed, and the secretary will send you daily bulletins, if a packet of addressed post-cards is supplied. I can tell you, on the patients' testimony, as well as on my own, that the nurses and doctors are kindness itself, and that no trouble and expense is spared—indeed, I have known of very bad cases in which a pound a day has been spent upon a patient—one, too, who could pay but three guineas for the whole course of treatment. Of course, there are elaborate arrangements for disinfecting the patients, as also their clothes, which are passed through a disinfecting machine, subjecting them to a heat of 360 degrees.

Provision for Convalescents.

"Female patients, and boys under twelve, can be sent to a scarlet-fever convalescent home, which despatches a special omnibus for them, thus enabling them to get a change of air before they are really fit to mingle with the outside world. But there is no such convalescent home for men as yet, though it is seen to be greatly needed, and would be at once built if funds were forthcoming.

"It may be just as well to point out that the fever hospital whose disgraceful administration was exposed some time ago, was of a perfectly different class to this, being, in fact, a rate-supported institution. The Secretary of the hospital whose work I have been here describing, tells me that, just lately, a lady, who, when those scandals came out, withdrew her promise of a legacy, quickly found out her mistake, and not only confirmed the legacy, but gave a handsome donation besides.

Prevention and Cure.

"I could give you many more details as to the management of this hospital, but space, I suppose, is limited, and, therefore, I will only say that it manifestly does a twofold work, its object being prevention as well as cure. Since its foundation it has treated 78,000 sufferers; but who can say how many thousands more it has saved from illness? The three guineas charged—except in special cases—to each patient, does not cover more than a quarter of the cost of his or her treatment; and, therefore, it has a strong claim upon all who benefit by it—that is to say, upon the general public."

### IV.—WITH THE CHILDREN.

By Mrs. Wilkins—a Mother.

"I do believe, sir, that there isn't one hour in the day or night that I haven't spent in a children's hospital, one time or another. You see I've had two or three children in, and they've always been bad, and bad for a long time, too, so that the doctors have "slated" them—that's to say, they've put them on the dangerous list, and then their friends could visit them any time, bless their poor little hearts! But there—it isn't about my own children you want me to tell, I know that.

"We Shouldn't Dare Touch 'em."

"And if I am to talk about hospitals, I can truthfully say I can't think where we should be without 'em. Why, even to people like me, as love their children, and make a happy home for 'em, a children's hospital is just the greatest help in the world. What ever could we do for a poor little mite that's got burnt, or that's been run over, or that's wasted away till it's nought but skin and bone—why, *we* shouldn't dare touch 'em! And then there's the doctoring, too, and the constant watching, and the operations—dreadful things, but they've saved many a life, I know that well enough. There's some folks 'll tell you '*they* won't go into hospital—they're not going to be pulled about by the doctors.' Well, but the doctors don't pull you about unless they can do you good by it—why should they? I remember once there was a little girl brought in who'd been run over by a tram in the street; she was torn right across, they said. The doctor came and looked at her, and then he put back the bedclothes and said, 'We'll just let her alone, nurse. It's no good worrying the poor little thing; she can't live more than an hour or so.' So the nurse just made her as comfortable as she could, and brought a baby-doll to see if she would like to have it to lie beside her; and the little thing smiled at it just like a mother smiles at her child, and then she put her cheek against its curly head, and went off to sleep, and never woke again.

For the Sake of the Children.

"Oh! it isn't to save ourselves trouble, sir, that mothers like me let our children go into hospital; it's because we know there's nothing else like it for them. I've seen some of the mothers nearly breaking their hearts over their children, and thinking of nothing but how they could be with them. I shall never forget the mother of little Maudie; she was the first to come, and the last to go, on visiting days. Maudie had hip-disease and had had to come into hospital, again and again, for months at a time. She was the only child, too, and her mother seemed as if she didn't know how to bear parting with her; but she always would do it, because she knew it was best for the child. And then there was another poor woman I'm thinking of; her little boy lay dying of consumption. When I saw him, he was that thin, you could see the skeleton behind his face; and she must have knowed he was dying, but she seemed as if she wouldn't think it. One day, I remember, she seemed to see it plain at last. She'd brought in a picture of him and the other boys playing in a band, and he wouldn't hardly look at it—he felt too bad, poor little chap. And then she burst out with a sudden cry like, 'Oh, my boy, my boy, and you looking so smart as you always did, and playing the big drum that beautiful! But I doubt you'll never play it no more.'

Cruel Parents.

"But when the poor little mites have got *cruel* parents—and there's a many that have—why, then the hospital's just 'Happy Land' to them. It makes your heart bleed to hear the poor little things sometimes, telling how they were scolded and beaten at home because they coughed, or because they couldn't help crying—their legs or their hips hurt them so. I've seen a poor little mite of six, with three great abscesses on his body, and arms and legs so thin you hardly dared touch him; and I've heard him tell, in his little weak whisper, how he'd crawl away into the cupboard when he heard father coming, because father beat him so.

"They Take the Poker to Me."

"And then there was Tim—I must tell you about Tim. He was a little chap of eleven, with little sticks of arms, and curly hair that fell over his face like a girl's fringe, and came down to his eyes—beautiful dark eyes they were that seemed to be put a little too forward. He'd got heart disease—the nurse told me, as well as bronchitis. It was she who told me first how he was beaten and 'tormented' at home, where there was a family of eight children, all living in the two rooms, besides a father dying of asthma, and a mother who went out charing. When I asked him how they tormented him, he told me that he would be lying asleep in a chair, and they would make a noise and wake him up, and then, he said, 'My temper gets over me, and I hit 'em, and then they hit me back again; they take the poker, to me sometimes.' 'Don't they love you?' I said; but the nurse broke in, 'I'm afraid he doesn't know what love means; and the worst of it is, it's the oldest that are most unkind to him. I'm told it's the sister of fourteen who knocks him about with a poker.' I asked him one day if he'd ever been to school, and he looked up at me so sadly, and said, "Yes, I used to go, but two years ago they took me off the books, because I couldn't get up the stairs.' Now, I ask you, sir, whatever would that poor little fellow do without a hospital?—and whatever does he do now?—for when I last went to that hospital, he was gone home. They can't keep those chronic cases right on, you know; it's not fair to all the others that are waiting. Of course he's cross sometimes—is it likely he wouldn't be, with his heart all wrong, and that crowd of children in the house?—and all the more for that, he wants to be somewhere where he'll be treated gently.

Maggie and Stevie.

"I could tell you of plenty more cases; when I begin I don't know where to stop. I've seen a little brother and sister brought in, almost starved, partly because they didn't have enough food, partly because they had bad digestions, and couldn't get any good out of bread and potatoes, which ain't fit food for a child, with nothing else put to 'em. They were as happy as could be in hospital, and after a fortnight you'd see 'em running about, and playing, and carrying messages for the other children 'all over the shop,' as they'd say. One day I said to little Stevie (he was only seven), 'Didn't you have enough to eat?' and he told me, very gravely, "No, mamma had no money. Father spent it to buy beer—that's naughty, that is. When I'm a man, I'm going to bring home all my money to mamma; and I've got some pennies now under my pillow—I'm going to give them to her when she comes, and she'll buy food for herself.' And in the same hospital, there was a poor baby that had got hurt by something the father had thrown at the mother; it was getting better when I saw it, but the eye was knocked clean out.

### Amateur Nursing.

"And sometimes the children get bad treatment at home, when their friends don't a bit mean it. There was a little boy came in one day with dreadful inflammation of his inside; and his two brothers who brought him said they didn't know what else to do—they'd promised his mother, who was gone, that they'd take care of him, and since he'd been ill they'd given him the best of everything, beefsteak puddings, and all he wanted! And I've seen another little boy who'd been fed in hospital on champagne, and I don't know what all, taken away by his friends—they would take him, though they were told it was very foolish. A letter came a day or two afterwards to say he had got home safely, and was given anything that he liked to eat; and in a day or two more, another letter came, to say that he was—dead!"

---

### V.—THE CHRONIC DISEASES OF CHILDREN; INCURABLE HOMES; AND OTHER SUPPLEMENTARY WORK.

By Miss Mather.

"I need hardly say that I fully sympathise with all that Mrs. Wilkins has said; and before I go on to my own special department, I may, perhaps, add that, to my mind, the value of children's—and, indeed, of all—hospitals is greatly enhanced by their moral effect. The cleanliness, the discipline, the great kindness, and yet the firmness, with which the patients are treated—these all leave indelible marks on the children, not to speak of the adults. A little rough, ill-mannered cub will leave the hospital gentle in manner, cleanly in his ways, and with a tongue trained to pretty childish language instead of to the foul words and oaths which poured forth when he came in. I don't mean, of course, to say that all have been badly brought up. Now and then we have little ones so amusingly and tiresomely virtuous that when the doctor asks them to put out their tongues, they flatly refuse, and cry out, 'Oh, no, no! My mother told me I was *never* to put out my tongue!' But there are only too many who seem to have had no training whatever, except in vice; and to these a hospital is not only a hospital, but a school, whose lessons are breathed in, like the air, in all unconsciousness.

### Chronic Cases.

"Of course I have had good opportunities of seeing this, having had so much to do with chronic cases. In such hospitals a child is kept for two, and even for four years, besides which they will often come in and out again several times. As you know, we relieve the hospital for hip cases, in which I am specially interested, as much as we can, and send home all cases which we think can be nursed by the mother—of course, with our supervision and advice. Sometimes the plan fails, as with a little patient re-admitted to-day, the child of a poor flower-woman, whose husband has been sent to prison once or twice for assaulting her and the child, the latter because it cried when it saw its mother knocked about. He has already tried to wreak his vengeance on the mother by taking the child away from her; and now that he will shortly be out of prison again we cannot but consent to take it in once more, in spite of the pressure upon the beds.

### "Cockles."

"You have taken a look round the wards? It must have struck you how bright and happy the children were, in spite of their being one and all on their backs. Perfect rest, amusement, and good food—these are our recipes; and very excellent ones they are, as returning health and never-failing spirits will show you. Of course, I am speaking of the majority of the cases, not, alas! of all. Did they show you Cockles, a tiny little fellow of three? He is always throwing his arms about, and rolling his curly head from side to side, to work off his spirits. It was he who taught the little Welsh girl to talk English; and it is the funniest thing to see how he 'bosses it' over Esther, in the next bed to his. She never even sings without first asking this youthful lord of creation, 'May I, Cockles?' Sometimes he tries—but in vain—to rouse her to some show of defiance, which, doubtless, he would promptly squash. 'Donkey!' he exclaims, 'Donkey—*donkey!*' And when she cannot be goaded into a retort, he turns round with quite a disappointed air, and says, 'She don't say nothing, do she?' But he is really tender-hearted; and one day, when the nurse was stooping over him, and her crucifix touched his shoulder, he stroked it softly, and whispered, 'Poor old Jesus!'

"Then, I dare say, they showed you our little patient from the Orange Free State, whose father saw the pictures of our hospital in the *Daily Graphic*, and brought his little invalid boy across the ocean to see if we could cure him.

### Winning the Little One's Hearts.

"One of the best evidences of the loving care with which the children are treated is their devotion to their doctors and nurses. They will count the hours when their own special nurse is away—I have heard a little boy say, in rather Biblical phraseology, I thought, to a nurse who had been off duty for a day, and only came in to wish him good-night, 'Oh, Nursie, where *have* you been? You've been gone all darkness and all light.' And they will beg the nurse to 'Tum down here,' and then put their little arms round her neck, and whisper, Now love me.' One little fellow in the hospital, announces that he is going to be a carpenter when he grows up, and live with Sister, and give her all his money; and as he is a deserted child, and she is devoted to him, I really think part of this may come true, if he ever lives to grow up. One of our little girls heard that a bandage was wanted for one of the doctors, who had hurt his hand, and thinking it was for her 'very own doctor,' she burst into piteous sobs, and when Sister hastened up, she wailed out (she cannot speak at all plainly), 'Oh Sitten—oh, my poor, poor docken!'

### Incurable Homes.

"I ought to be able to tell you something about Incurable Homes, too, for I was a constant visitor at one of them before I took up this regular work. But I really think no word from me is needed to demonstrate their usefulness. To send a poor little Incurable back into a dirty, noisy, crowded house, where the parents are ignorant, rough, and, perhaps, even cruel, is manifestly—I was going to say impossible, but, alas! want of funds too often forces such a course upon us. Take a child with bones so brittle that, even in hospital, various fractures have occurred, eight weeks being the longest time on record in which no bone has been broken; or a child with incurable heart disease; or one so terribly malformed that it can only lie in one position—on its stomach; and say—what can we do for these poor little things but give them a home where they will be tenderly nursed and cared for, so long as their sad little lives shall last?

Samaritan Funds.

"Then I am to say a word about Samaritan Funds. Well, I think most people will agree with me that there is hardly any part of the work of a hospital more important than that done by these funds, when I say that it is to them the patients must look, when they leave the hospitals, weak and worn, for help to get surgical appliances; for tickets for an Invalids' Dinner-table; for warm clothing; for new milk (if they are children); for money to keep them going (if they are breadwinners) till they are strong enough to take up work again; and best of all, for tickets for convalescent homes. Without some such helps as these, the work done by the hospitals is very likely to be all undone again, as in the case of a poor little boy whom I know very well.

Living on Parsnips.

"Walter left the hospital to come back to a wretched house, where the father was at home all day, having been out of work for a long time; for two whole days, as I know for a fact, the family had nothing at all to eat but some parsnips, kindly given them by a neighbour; and for weeks, food was terribly scarce with them. What wonder that that poor little fellow is now in hospital again? And yet, I ask you, are *we* to blame for such things? Can we *make* money? Oh! how one longs sometimes for the age of miracles to return (and yet *ought* one to need a miracle?)—for coffers which, like the cruse of oil, 'shall not fail!'

Convalescent Homes.

"And Convalescent Homes? Well, we know that no rich man thinks it possible to get strong again after a bad illness, without a week or two in the country or at the seaside; and what applies to him applies with tenfold force to the dwellers in our crowded streets and alleys. I don't know of anything much more sad than the simple little statement which I have more than once seen in the report of a hospital: 'The convalescent home has been closed for want of funds.' Looked at in the abstract, this does not seem to mean so very much, but looked at in the concrete, it simply means the sending back to their poverty-stricken homes men who are too weak to work; women to whose shaken nerves the noise and worry of the household is almost insupportable; children whose little frames, wasted and worn by sickness, cry out for the tonic of the sea-air, and the restorative of good and sufficient food. Of course, these hospitals do what they can to secure beds at other convalescent homes; but those who know how sadly the demand for letters exceeds the supply, are only too well aware that the success of one poor patient—a success for which he has constantly to wait for four or six weeks—only means the failure of two or three more.

"Restored to Life and Power and Thought."

"I have just been looking at a batch of letters received by the Matron of an excellent convalescent home, and more grateful and appreciative letters I have never seen. With the women and children, they seemed to be but a continuation of what had been said before by word of mouth; with the men they were sometimes, at any rate, the first outbursts of gratitude, for, as one remarks, 'I couldn't stop to talk to you the morning I left; if I had, it might have upset me, and I did not wish to show it.' Very characteristic of a man, don't you think so? But the gratitude will out at last; and how, indeed, should it not, when one thinks of all that restored health and vigour really mean to a man who has to work hard for his daily bread and for the daily bread of all his dear ones?

A Convalescent Hospital.

"There is much, very much, more that I could say, when I am on this subject. I could tell, for instance, of a lady who has lately started a little Convalescent Hospital, rather than Home, in her own village, to which come children who need strengthening up before or after serious operations, and whose cases necessitate careful surgical nursing. I only wish that other ladies would follow her example. They would find, as she does, that the keeping up of such a place is by no means incompatible with the many and varied occupations and interests of home life; and to ladies who find their lives somewhat empty and colourless, work of this kind would be a constant source of interest,—a godsend, in fact, to them as well as to the children.

THE END.

